# Endothelial Cell Therapy for the Acute and Chronic Liver Disease

**DOI:** 10.1055/a-2841-9824

**Published:** 2026-04-09

**Authors:** Dilnar Mahmut, Valerie Gouon-Evans

**Affiliations:** 1 Section of Gastroenterology, Department of Medicine, Center for Regenerative Medicine, Boston University Chobanian and Avedisian School of Medicine, Boston Medical Center, Boston, Massachusetts, United States

**Keywords:** endothelial cell therapy, liver sinusoidal endothelial cell, vascular targeted liver regeneration, capillarization, liver disease

## Abstract

Endothelial cell dysfunction and loss are key drivers of acute and chronic liver disease, underscoring a critical unmet need for liver vascular–targeted therapies. Liver sinusoidal endothelial cells are specialized endothelial cells that form a fenestrated microvascular niche regulating hepatocyte metabolism, immune homeostasis, and hepatic stellate cell activation. Repopulating this niche through transplantation of primary liver sinusoidal endothelial cells, endothelial progenitor cells, induced-pluripotent stem cell–derived liver sinusoidal endothelial cells, or engineered endothelial cells delivered within biomaterial aims to restore microvascular architecture, reestablish supportive angiocrine signaling, attenuate fibrosis, and promote liver regeneration. This review summarizes the biological rationale for endothelial cell–based therapy, compares cell sources and engineering strategies, evaluates cell delivery and engraftment approaches, and synthesizes preclinical evidence demonstrating therapeutic benefits across diverse animal models of liver injury. Finally, we highlight key translational challenges and propose future directions to accelerate the clinical development of endothelial cell–based therapies for liver disease.

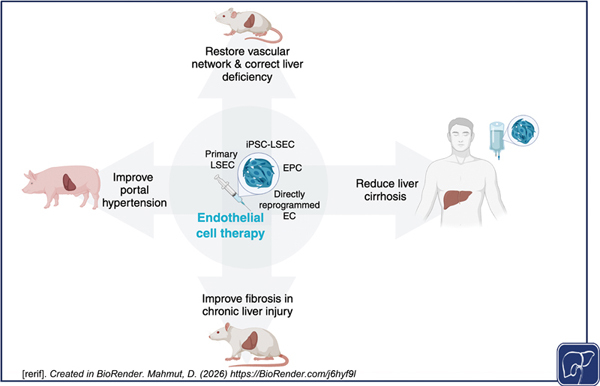

## Introduction

The liver possesses a highly specialized vascular system designed to support its central roles in metabolism, detoxification, and immune surveillance. Unlike most organs, the liver receives a dual blood supply from the portal vein and the hepatic artery. The portal vein delivers nutrient-rich, gut-derived blood, while the hepatic artery supplies oxygenated systemic blood. These two inflows converge within the hepatic sinusoids—low-pressure, highly permeable capillary channels that traverse the liver lobule and drain into the central vein, ultimately returning blood to the systemic circulation via the hepatic veins.

Liver sinusoidal endothelial cells (LSECs) are a specialized endothelial population lining the hepatic sinusoids, forming a discontinuous, fenestrated barrier that lacks a basement membrane. 1 2 Their fenestrae (50–150 nm pores) are organized into sieve plates, collectively forming the “liver sieve,” which enables rapid exchange of plasma solutes, nutrients, lipoproteins, and metabolites between sinusoidal blood and the space of Disse. 3 This unique architecture confers the highest permeability of any mammalian endothelium and underpins key LSEC functions, including filtration, scavenging, and immune surveillance. 4 5 LSECs express a diverse array of scavenger receptors, including stabilin-1, stabilin-2, the mannose receptor, and FcγRIIb, which mediate the clearance of circulating macromolecules, waste products, and immune complexes. Through these mechanisms, LSECs account for up to 45% of the liver's total pinocytic activity. 4 5 Given their unique and complex structure, LSECs play a critical role as an instructive cellular niche during liver development and regeneration. The essential role of liver ECs was first demonstrated in studies of the developing murine liver, which showed that vasculogenic ECs and nascent vessels are required for the earliest stages of liver organogenesis, prior to the onset of functional blood flow. 6 Using pluripotent stem cell (PSC) differentiation systems directed toward the hepatic lineage, subsequent studies revealed that ECs function as a niche by repressing Wnt and Notch signaling pathways to promote hepatocyte specification. 7 8 This supportive effect depends on activation of VEGFR2 expressed on ECs. 9 In the adult liver, mature LSECs perform a broad array of homeostatic functions. They produce nitric oxide (NO) to regulate intrahepatic vascular tone and maintain hepatic stellate cell (HSC) quiescence, 10 11 and they secrete angiocrine factors, including Wnt2, Wnt9b, and hepatocyte growth factor (HGF), that drive hepatocyte proliferation, β-catenin–mediated metabolic zonation, and liver regeneration. 12 13 14 Overall, LSECs demonstrate significant spatial heterogeneity and perform distinct, specialized functions across the liver lobule, playing a critical role in maintaining hepatic homeostasis. 11 15 16 17 18 19 In this review, we focus on the primary endothelial dysfunction that underlies acute and chronic liver injury, while acknowledging that disease-specific alterations have also been described in metabolic-associated steatotic liver disease (MASLD), alcohol-related liver disease (ALD), and hepatocellular carcinoma (HCC). 20 21 22 23 24

During acute liver injury, LSECs deteriorate rapidly, preceding and amplifying hepatocyte damage. In acetaminophen-induced toxicity, for example, LSECs are direct targets of reactive metabolites, exhibiting cellular swelling, intercellular gap formation, and hemorrhage into the space of Disse within hours of overdose, events that precede zone 3 hepatocyte necrosis. 25 This early microvascular collapse disrupts sinusoidal perfusion and nutrient exchange, thereby exacerbating hepatocellular injury. 25 Strain-dependent differences further indicate that some LSECs are directly susceptible to drug metabolites through cytochrome P450–mediated pathways, whereas others are injured indirectly via hepatocyte-derived toxic intermediates. 26 In ischemia–reperfusion injury (IRI), cold preservation of the liver leads to ATP depletion and cytoskeletal disruption, resulting in LSEC rounding and detachment. 20 Subsequent reperfusion triggers oxidative stress and excessive reactive oxygen species generation, while downregulation of endothelial nitric oxide synthase (eNOS) reduces NO production and promotes endothelial dysfunction. 20 27 Concurrently, activated Kupffer cells release proinflammatory cytokines such as TNF-α and IL-1β, inducing upregulation of adhesion molecules (ICAM-1, E-selectin, and P-selectin) on LSECs. This promotes neutrophil recruitment, platelet aggregation, and microthrombosis, further aggravating microvascular injury. 20 Following partial hepatectomy, excessive portal shear stress in small-for-size liver remnants can cause endothelial denudation, sinusoidal congestion, and hemorrhagic necrosis. 28 Experimental disruption of VEGFR2 signaling in LSECs impairs early hepatocyte proliferation, highlighting the importance of VEGFR2-mediated angiocrine factors, including HGF and Wnt2, secreted by LSECs for effective liver regeneration. 6 29 30 Moreover, early capillarization of LSECs, characterized by fenestrae loss and basement membrane deposition, further disrupts LSEC–hepatocyte crosstalk, promotes hepatic stellate cell activation, and facilitates progression from acute injury to chronic fibrosis. 11 20

During chronic liver injury, LSECs undergo progressive phenotypic alterations that drive fibrogenesis and portal hypertension. 20 21 31 Capillarization, the hallmark of LSEC dysfunction in chronic disease, is characterized by loss of fenestrae, deposition of basement membrane components such as collagen IV and laminin, and upregulation of continuous endothelial markers including CD34 and CD31. 20 26 31 Single-cell transcriptomic analyses reveal that zone 3 LSECs are particularly vulnerable in cirrhosis, exhibiting marked downregulation of LSEC-specific markers (Lyve1, Cd32b, Flt4, Stab2) alongside several-fold upregulation of CD34 expression. 20 Notably, capillarization precedes fibrosis in both human cirrhosis and experimental mouse models, supporting a causal role in disease progression. 20 21 Mechanistically, capillarized LSECs lose VEGF-mediated NO production and fail to maintain HSC quiescence. 17 Key transcription factors required for eNOS expression, including KLF2, KLF4, and AP-1 components (Fos, Fosb, Jun, Junb), are downregulated across all three zonal LSEC populations, contributing to increased intrahepatic vascular resistance and portal hypertension. 20 Dysfunctional LSECs actively promote fibrogenesis through: (1) secretion of profibrotic angiocrine factors (TGF-β, PDGF, hedgehog ligands, excess VEGF) that activate HSCs 20 21 29 32 33 ; (2) partial endothelial-to-mesenchymal transition (EndMT) characterized by co-expression of endothelial (CD31) and mesenchymal (α-SMA) markers and deposition of extracellular matrix (fibronectin, collagens) preferentially in sinusoidal regions 2 34 ; and (3) altered mechanosensing associated with Notch1-Piezo1 signaling driving CXCL1 secretion, neutrophil recruitment, NETosis, and microthrombosis under increased shear stress. 20 Chronic injury shifts angiocrine signaling from regenerative (CXCR7-Id1–mediated HGF/Wnt2 release) to fibrogenic (CXCR4-driven TGF-β/BMP2/PDGF-C) secretion. 29 In parallel, LSECs lose scavenger function through downregulation of endocytic receptors (Mrc1, Stab1, Stab2, Scarb1, Scarb2, Fcgr2b), especially in zone3, impairing clearance of immune complexes, waste products, and pathogens. 20 21 35

Despite growing evidence that LSEC dysfunction represents an early and potentially reversible event preceding fibrosis, substantial therapeutic gaps remain. 20 First, the lack of highly specific LSEC markers and promoter-driven Cre mouse models limits rigorous mechanistic studies and target validation, although the recently discovered Oit3 marker for LSEC is promising. 20 36 Pharmacologic compounds such as simvastatin, lanifibranor, YC-1, and hedgehog pathway inhibitors demonstrate anti-capillarization and anti-fibrotic effects in rodent models, primarily through restoration of fenestration or eNOS-sGC signaling; however, none have advanced to clinical trials specifically as LSEC-targeted therapies. 2 17 20 21 31 Second, current delivery platforms lack sufficient LSEC specificity. Nanoparticle systems targeting Stab2 or hyaluronan receptors are limited by extrahepatic expression, thereby compromising selectivity and therapeutic precision. 36 The recent identification of Oit3 as a promising LSEC-restricted hallmark gene could enable more precise targeting; however, translation to clinical application remains distant. 36 Third, the optimal therapeutic window remains poorly defined. Capillarization arises early during fibrogenesis, when intervention may be most effective, yet most patients present at advanced stages of disease, at which point basement membrane deposition and structural remodeling may be less reversible. 2 17 20 31 Fourth, the molecular drivers of LSEC zonation and upstream regulators of the master LSEC transcription factor GATA4 remain incompletely understood, limiting the development of rational, mechanism-drug based therapies. 17 Finally, it remains unclear whether restoration of the LSEC phenotype in established cirrhosis can reverse fibrosis or primarily halt further progression. Moreover, LSEC-directed therapies have not yet been systematically evaluated in combination with emerging antifibrotic agents or immunotherapies. 20 21

**Fig. 1:**
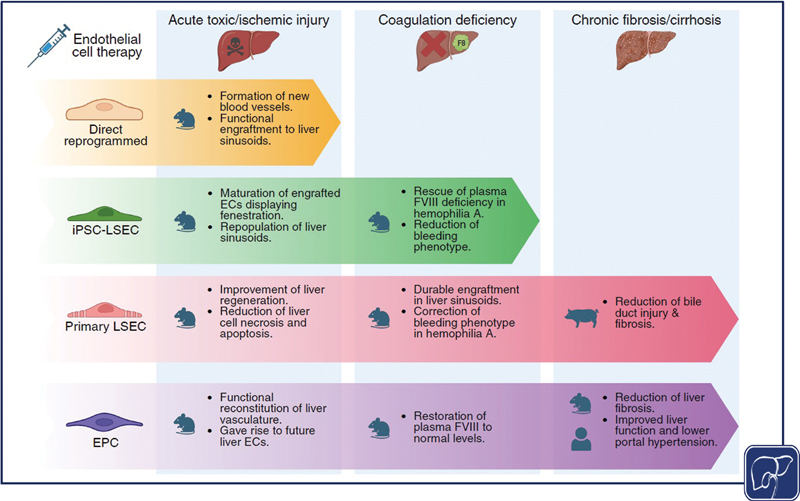
Endothelial cell (EC) therapy from different cellular sources to improve liver disease outcomes. In mouse models that recapitulate partial hepatectomy and acute drug-induced liver injury, engraftment of ECs into the sinusoids improves liver regeneration and restores the vascular network. Transplantation studies using primary (primary LSECs) and iPSC-derived LSECs (iPSC-LSEC), as well as endothelial progenitor cells (EPCs), to correct the bleeding phenotype in hemophilia have demonstrated restoration of plasma factor VIII (FVIII; F8) levels to near-normal levels in treated mice. Additional preclinical studies evaluating EPCs have reported moderate improvements in fibrosis associated with chronic and cirrhotic liver disease in rodent and porcine models, with early evidence of potential benefit in human studies. [rerif]. *Created in BioRender. Mahmut, D. (2026)* https://BioRender.com/1z1cnpe

EC therapy offers a compelling approach for liver disease by directly addressing LSEC dysfunction, which extends beyond passive blood transport ( Fig. 1 ). LSECs secrete angiocrine factors essential for liver regeneration, produce coagulation factors including factor VIII (F8), and regulate hepatocyte function through paracrine signaling. 37 38 39 40 In chronic liver disease and fibrosis, pathological capillarization disrupts these functions, leading to HSC activation, increased vascular resistance, and impaired hepatocyte renewal. 1 14 20 21 31 41 Transplantation studies demonstrate that healthy ECs can engraft liver sinusoids, restore a differentiated LSEC phenotype with functional fenestrations, and correct metabolic deficiencies. 38 42 43 Matrix-embedded ECs are particularly promising, as biomaterial scaffolds protect transplanted cells from immune rejection while preserving their reparative secretome. 44 45 46 47 Proof-of-concept studies in mouse models indicate that EC transplantation reduces fibrosis markers, improves liver function, and modulates inflammatory responses toward regenerative phenotypes. Specifically, in hemophilia A mouse models, engrafted LSECs restore F8 to therapeutic levels. 38 42 48 The ability to generate functional LSECs from human pluripotent stem cells provides a scalable source for cell therapy, 37 39 positioning EC therapy as an integrated approach capable of restoring both the vascular niche and parenchymal function in liver disease.

## Preclinical Evidence

### Primary LSECs

Primary LSEC transplantation studies demonstrate robust therapeutic potential across multiple liver injury models ( Table 1 ). Transplanted LSECs engraft liver sinusoids, integrate into the endothelial layer between hepatocytes, and maintain a differentiated phenotype characterized by fenestrations and active scavenger receptors. 38 Engraftment efficiency is markedly enhanced by sinusoidal preconditioning; for example, monocrotaline (MCT) treatment increases donor cell incorporation from minimal levels to several percent of non-parenchymal cells over months, enabling long-term survival without redistribution to extrahepatic organs. 38 In hemophilia A models, primary human fetal LSECs transplanted into uPA-NOG mice secrete human F8 at near-physiologic levels, achieving therapeutic correction (>10% F8 activity) without inducing inhibitory antibodies. 38 43 Cultured primary LSECs retain transplantation capacity and, when combined with hepatocytes and other cell types, can contribute to multilineage liver grafts. 49 Matrix-embedded primary ECs provide additional benefits in ischemic injury models: they form functional vascular anastomoses, markedly reduce DNA fragmentation and apoptosis in ischemic lobes, and promote liver regeneration by shifting macrophages from pro-inflammatory M1 to regenerative M2 phenotypes while upregulating HGF to recruit endothelial progenitors. 44 In a large-animal cirrhosis model, catheter-directed intraportal infusion of autologous CD31 ^+^ primary LSECs, isolated from each pig's liver biopsy and briefly expanded ex vivo, proved technically feasible and safe, and resulted in cell persistence without portal vein thrombosis. 48 Although reductions in fibrosis and biliary proliferation did not reach statistical significance, this study established a translational platform for primary LSEC transplantation in large animals. 48 Collectively, these studies demonstrate that primary LSECs can restore sinusoidal architecture, correct endothelial-dependent metabolic defects, and improve outcomes in both acute and chronic liver injury.

**Table 1:** Transplantation studies testing preclinical efficacy of EC therapy in liver disease

Cell therapy	Disease model	LSEC source	Delivery route	Recipient animal	Key outcomes	Engraftment efficiency	Mechanistic insights	Ref.
Primary LSECs	HemophiliaA (F8 −/− ) mice with monocrotaline pre-condition	Primary mouse LSECs from Tie2-GFP FVB/N donors	Intrasplenic injection	Immunodeficient F8 −/− FVB/N mice	Durable repopulation of liver endothelium; plasma F8 ≈19% of normal; full correction of bleeding phenotype	Up to approximately 9% of non-parenchymal liver cells GFP ^+^ LSECs at 3months with MCT preconditioning	MCT selectively injures host LSECs, giving transplanted cells a proliferative advantage; graft cells re-establish sinusoidal phenotype and FVIII production, demonstrating LSEC as principal F8 source	35
	uPA-NOG hemophiliaA mice	Human fetal liver LSECs purified from mid-gestation liver	Intrasplenic injection	Immunodeficient uPA-NOG mice with humanized liver niche	Human F8 in plasma up to approximately 30% of normal human levels; human LSECs lining mouse sinusoids in long term	Long-term persistence; no extra-hepatic redistribution	Transplanted human LSECs localize to sinusoids and maintain LSEC marker profile, confirming they are a major human F8 source in vivo	40
	Porcine transarterial ethanol–iodized oil embolization	Autologous porcine liver ECs	Catheter-directed intraportal infusion	Yorkshire porcine with established cirrhosis	Trends toward less fibrosis, bile duct injury, and higher hepatocyte proliferation, but not statistically significant (pilot *n* = 4 + 4)	ECs detectable in liver at 1h by FACS and RFP labeling; no durable engraftment at 3weeks (RFP ^+^ cells undetectable)	Benefits likely mediated by short-term angiocrine signaling (e.g., VEGF, angiopoietin-2) rather than permanent engraftment; study establishes catheter-directed intraportal EC delivery platform for translation	45
	70% partial hepatectomy in mouse	Healthy HUVECs (some GFP-tagged) embedded in compressed collagen (Gelfoam) as matrix-embedded ECs (MEECs)	Surgical placement of MEEC-loaded collagen scaffold bridging host median lobe and graft	C57BL/6 mice undergoing hepatectomy plus graft implantation	MEECs formed a functional “vascular splice” bridging host and graft; reduced congestion, ischemic necrosis, apoptosis, and inflammation; improved liver regeneration (approximately 15% higher mass) and function; reduced rejection in allografts without immunosuppression	Not quantified (embedded cells remained locally)	MEECs secrete pro-angiogenic and immunomodulatory factors, recruit EPCs via CXCR4/CXCR7, shift macrophages from M1 to M2, increase HGF and other trophic signals, thereby rescuing dysfunctional endothelium and promoting hepatocyte survival and engraftment	38
Endothelial progenitor cells (EPCs)	CCl _4_ -induced liver cirrhosis in male 6-wk Wistar rats	Bone-marrow-derived EPCs isolated from donor rats	Weekly tail-vein injection (4weeks)	Male 6-wk Wistar rats	↓ portal venous pressure (10.8 ± 1.0 vs. 14.6 ± 0.8 mmHg) ↑ hepatic blood flow (12.7 ± 1.9 vs. 7.3 ± 1.0 mL min ^−1^ 100 g ^−1^ ) ↓ fibrosis area (5.2 ± 1.3 % vs. 10.8 ± 2.2 %) ↑ sinusoidal vascular density (eNOS ^+^ vessels)	PKH26-red-labeled EPCs detected around portal tracts, fibrous septa, lobules; EPCs CD31 ^+^ and PCNA ^+^ /isolectin B4 ^+^ cells increased, indicating incorporation into sinusoids	EPCs up-regulate p-eNOS and VEGF, enhancing NO production; downregulate endothelin-1 and ET-A receptor mRNA; reduce caveolin expression; promote angiogenesis and sinusoidal re-endothelialization, thereby lowering intra-hepatic resistance and fibrosis	47
	Decompensated liver cirrhosis (Child-Pugh/ MELD ≈ 10–16) in humans	Autologous bone-marrow-derived EPCs cultured 7 days	Hepatic artery infusion	Human patients (n ≈ 11 treated)	Safe and feasible (91% feasibility); significant MELD score drop at 90 days (P = 0.047) and HVPG reduction in 5/9 survivors; two patients delisted for transplantation	No labeled cells; engraftment not quantified	Clinical benefit correlated with higher percentages of EPCs expressing VEGFR-2, vWF and acLDL (R = 0.87 for vWF versus HVPG change); trend toward greater secretion of HGF, SDF-1, and IGF-1 and lower IL-6 suggests paracrine modulation of fibrosis and vascular tone; likely mediated via NO/VEGF pathways	48
	MCT-induced acute & hemophilia A mouse models	Syngeneic transplant of CD157 + CD200 + EGFP+ enriched vascular endothelial stem cells (VESCs)	Intrahepatic & intrasplenic injection	MCT + irradiated C57BL6J & hemophilia A mice	CD157 + CD200+ functionally reconstituted liver vasculature; plasma F8 rose from <1% (untreated) to 62.9% of normal	In single-cell assays, 3/350 CD157 ^+^ CD200 ^+^ cells produced ≥592 EC progeny each and full vascular trees; engraftment was represented qualitatively	Lineage tracing shows CD157 ^+^ CD200 ^+^ VESCs continuously replace endothelial cells of large vessels and sinusoids for more than a year under steady-state conditions, and they expand segmentally after acute injury, supplying tip, stalk, and phalanx cells that form new vessels.	19
iPSC-derived LSECs	Drug induced acute liver failure model in immunodeficient Alb-Tk-NOG mice	hiPSC-liver bud: hiPSC-derived hepatic endoderm hiPSC-derived ECs hiPSC-derived stromal cells (iPSC-STM)	Subcapsular renal transplantation	Alb-Tk-NOG mice	Buds vascularized within 48 h, formed functional anastomoses; human albumin persisted >15 weeks; improved survival vs. sham (≈40 % vs. 20 % at day 50)	Not reported as a numeric engraftment %; functional vascularization and long-term survival were demonstrated instead	Co-culture of iPSC-EC and iPSC-STM is required for organ-bud vascularization; VEGF-Notch-modulated differentiation generates functional endothelial progenitors; transplanted buds self-organize, recruit host vessels, and achieve mature hepatic gene expression	50, 52
	Neonate NSG Adult NSG (Monocrotaline injury)	hESC/PSC-derived angioblast & LSEC-like cells (LSEC-LCs)	Neonate: intra-hepatic injection; Adult: intrasplenic injection	NSG mice – neonatal (P1–P4) and adult (6–10 weeks, MCT-conditioned)	Higher efficiency of human cell engraftment in adult NSG-MCT model compared with NSG neonates. Engrafted cells displayed fenestrated sieve plates, robust Ac-LDL/ *E. coli* uptake	Neonatal venous angioblasts: approximately 47% of mice showed >1% engraftment; Adult MCT-conditioned mice: 100% of mice showed >1% engraftment, with up to approximately 40% of non-parenchymal cells being RFP ^+^ CD31 ^+^ LSEC-like	Venous lineage preferentially yields LSECs (venous angioblasts engraft more efficiently than arterial); specification modulated by VEGF-Notch dosage (low VEGF + Notch inhibition favors venous fate); hypoxia/HIF-1α and cAMP signaling enhance LSEC-LC maturation; WNT2-mediated crosstalk promotes hepatocyte–endothelial interaction	34
	Hemophilia A (NSG-HA mice lacking F8)	hPSC-derived venous endothelial progenitors (VECs)	Intrasplenic injection	NSG-HA mice	Sustained human FVIII (approximately 15% of normal) up to 17 wk; bleeding time normalized; VECs matured into CD31 ^+^ CD32 ^+^ LYVE-1 ^+^ LSECs; competitive assays showed 10–20-fold higher engraftment vs. earlier transplantation studies	Engraftment reached 38.6 ± 1.5% of non-parenchymal cells by day 100 (≥1% NPC threshold met in all mice); early (1 day) engraftment approximately 0.14 % → expansion over time	Mesoderm induction (12 ng BMP4 + 4 ng Activin A) is the critical determinant of engraftment efficiency (10–20× better than prior protocol); arterial/venous specification method has minimal impact; CD235a/b ^+^ mesoderm subset correlates with high-engraftment VECs	39
	Fah ^−^ / ^−^ /Rag2 ^−^ /-/Il2rg ^−^ /- (FRG) mouse with NTBC-cycling liver injury-regeneration cycles	hiPSC-derived CD31 ^+^ endothelial cells (iECs) (tdTom/eGFP-labeled)	Intrasplenic injection	FRG mice	Progressive sinusoidal repopulation; human FVIII ≈11% of normal plasma at 12 wk; acquisition of LSEC markers (CD14, CD36, LYVE-1, STAB2) and zonation (Zone 1 → Zone 3)	Peak at 4 wk: 10.6 ± 3.7% of liver vasculature; 2.7 ± 1.1% (1 wk) → 4.9 ± 2.2 % (2 wk) → decline by 12 wk	Liver micro-environment drives transcriptional shift to LSEC fate (↑NOTCH1, GATA4, MAF, EMCN, CLEC14A); VEGF-Notch circuit and hypoxia-responsive genes promote sinusoidal specification and fenestration	55
	Hemophilia A (F8-deficient plasma; NSI-FVIII KO mice)	Human liver bud organoid (HLBO) containing hiPSC-LSEC progenitor (iLSEP)	Orthotopic transplantation to the liver	NSI-FVIII KO mice	HLBOs with iLSEPs formed fully perfused, sinusoid-like human vessels (70 kDa dextran permeable, 2 MDa excluded); rescued F8 deficiency, shortened APTT, restored thrombin generation, reduced bleeding for ≥5 mo	Engraftment demonstrated qualitatively (human vessels anastomosed to host, persisted ≥5 mo); no quantitative % engraftment reported	WNT2 secreted by iLSEPs drives sinusoidal-to-hepatocyte crosstalk, enhancing hepatocyte differentiation and endothelial branching; OSM signaling (dose-dependent) induces iLSEP → iLSEC maturation; knock-down of WNT2 reduces endothelial network density; functional sinusoid characteristics confirmed by dextran permeability and Ac-LDL uptake	36
Directly reprogrammed ECs	Ischemia of hind limbs	Human fibroblasts directly reprogrammed to EC-like cells using pluripotency factors (PiPs-Ecs)	Intramuscular injection	SCID mice	PiPS-ECs markedly accelerated blood-flow recovery; significantly higher foot-blood-flow ratios at both 1 week (≈0.7 vs. ≈0.55 for fibroblasts) and 2 weeks (≈0.9 vs. ≈0.7) post-ischemia; approximately 2-fold increase in capillary density (≈280 vs. ≈175 per mm ^2^ ) compared with fibroblasts	Human-specific CD31 staining demonstrated an approximately 120-fold rise in engraftment of PiPS-ECs versus fibroblasts	Reprogramming required introduction of pluripotency factors; in their absence fibroblasts did not convert to EC-like cells	56
	70% partial hepatectomy in mouse	Human mid-gestation c-Kit ^+^ amniotic cells directly reprogrammed to rAC-VECs	Intrasplenic injection; subcutaneous injection of Matrigel plugs	NSG mice	rAC-VECs formed numerous functional, perfused vessels in Matrigel plugs and re-vascularized regenerating liver sinusoids	Approximately 5–10% of regenerated liver sinusoidal vessels were Isolectin ^+^ hCD31 ^+^ rAC-VECs	Optimal stoichiometry of ETS factors (transient ETV2, constitutive FLI1/ERG1) plus 3-week TGFβ inhibition “locks in” a stable vascular program, permanently functionalizes VEGFR2 signaling, and yields highly proliferative, mature rAC-VECs that maintain EC identity upon serial passaging and in vivo engraftment	57

Abbreviations: HVPG, hepatic venous pressure gradient; MELD, model for end-stage liver disease; NPC, non-parenchymal cell.

Note: *Engraftment efficiency is reported when quantitative data are available in the source material; “N/A” denotes that the study did not provide a numeric estimate.

### Endothelial Progenitor Cells

Endothelial progenitor cell (EPC) therapy combines readily obtainable bone marrow–derived EPCs, which express high levels of pan-endothelial markers while lacking hematopoietic markers, 50 51 including a rare CD157 ^+^ tissue-resident vascular endothelial stem cell (VESC) population that exhibits long-term self-renewal and the capacity to repopulate all hepatic vascular compartments 52 ( Table 1 ). Together, these populations provide complementary mechanisms for vascular regeneration and functional restoration. Bone marrow–derived endothelial progenitor cells (BM-EPCs) represent a relatively mature endothelial population, with reduced progenitor marker expression (CD133, CD34) and high expression of functional EC markers including CD31, KDR/VEGFR-2, and vWF, along with robust acetylated-LDL uptake, while remaining essentially negative for hematopoietic markers (CD45, CD68). 50 51 In carbon tetrachloride (CCl _4_ )-induced cirrhotic rats, weekly EPC infusion for several weeks significantly reduces portal venous pressure and increases hepatic blood flow compared with saline-treated controls. 50 BM-EPC transplantation decreases liver fibrotic area, increases sinusoidal endothelial density, and expands PCNA ^+^ /isolectin B4 ^+^ ECs, indicating vascular reconstitution. 50 These improvements are accompanied by upregulation of eNOS and phospho-eNOS, increased hepatic VEGF, reduced endothelin-1 levels, and fewer α-smooth-muscle-actin-positive myofibroblasts. 50 A subsequent phase 1 and 2 trial in patients with decompensated liver cirrhosis evaluated autologous BM-EPCs delivered via the hepatic artery. 51 The procedure was feasible in most patients and demonstrated a favorable safety profile, with no treatment-related severe adverse events, portal vein thrombosis, embolic complications, or immune-mediated rejection over 1 year of follow-up. 51 Model for end-stage liver disease (MELD) scores improved significantly at 90 days, and most surviving patients demonstrated reduced hepatic venous pressure gradients. Notably, therapeutic benefit correlated with BM-EPC functional quality rather than cell dose: higher proportions of VEGFR-2 ^+^ , vWF ^+^ , and acLDL-uptaking cells were associated with greater improvements in liver function and portal hemodynamics. 51 Mechanistically, BM-EPCs appear to act largely through paracrine effects, secreting hepatoprotective factors such as HGF and IGF-I, with delayed improvements in portal hypertension suggesting indirect modulation of resident liver cells rather than direct endocrine effects. 50 51 Collectively, these preclinical and early clinical studies support BM-EPC transplantation as a safe and feasible strategy for cirrhosis, with efficacy mediated by vascular repair, hemodynamic improvement, and attenuation of vasoconstrictive mediators.

Recently, CD157 (BST1) was identified as a surface marker defining a rare population of tissue-resident VESCs residing in large arteries, veins, and portal vessels of adult mice. 19 These CD157 ^+^ cells display clonal colony-forming ability, long-term self-renewal, and the capacity to regenerate all vascular compartments of the liver. 19 To evaluate their therapeutic potential, CD157 ^+^ CD200 ^+^ VESCs were isolated, expanded in vitro, and transplanted into a murine hemophilia A model. At 6 weeks post-transplantation, plasma F8 activity rose from <1% in untreated controls to 62.9% of normal (range 57.4–66.7%), and hepatic F8 mRNA levels increased approximatively three-fold, demonstrating that the donor-derived VESCs engrafted as functional ECs were capable of synthesizing and secreting F8. 19 In a tail-clip bleeding assay, bleeding time, which exceeded 60 minutes in untreated mice, was shortened to 5.6 ± 1.1 minutes in VESC-treated animals, confirming phenotypic rescue of the bleeding disorder. 19 Histological analyses revealed extensive donor-derived GFP+ vessels throughout the liver, including portal veins, sinusoids, and central veins, with restored fenestrations and proper basement membrane composition, indicating full structural and functional integration of the transplanted VESCs. 19 These findings establish CD157 ^+^ VESCs as a highly potent endothelial progenitor population capable of both vascular regeneration and correction of a systemic coagulation defect.

### iPSC-derived LSECs

The first demonstration that iPSC-derived ECs can support functional liver tissue came from studies in which iPSC-derived hepatic endoderm (iPSC-HEs), human umbilical vein endothelial cells (HUVECs), and mesenchymal stem cells spontaneously assembled into three-dimensional liver buds (iPSC-LBs) within days, recapitulating key organogenetic interactions 53 54 ( Table 1 ). Following transplantation into immunodeficient mice, these liver buds rapidly connected to host vasculature, with donor ECs forming functional anastomoses as confirmed by intravascular tracer studies. 53 The grafts exhibited liver-specific functions, including albumin secretion and human-specific drug metabolism, and mesenteric transplantation rescued otherwise lethal drug-induced liver failure. 53 Building on this platform, a subsequent protocol generated liver buds entirely from iPSCs by differentiating hepatic endoderm (iPSC-HEs), ECs (iPSC-ECs), and septum transversum-like mesenchyme (iPSC-STM) using staged BMP4/GSK-3β inhibition followed by VEGF-A/Notch modulation. 55 This approach yielded large numbers of endothelial progenitors within days, expandable to tens of millions of mature ECs that formed patent vessels after subcutaneous transplantation, and improved cardiac function when delivered into ischemic myocardium. 55 56

A major advance in generating liver-specific endothelium was achieved when venous endothelial cells (VECs), derived from BMP4/activin A-induced KDR ^+^ CD235a/b ^+^ mesoderm, were shown to engraft the liver and adopt LSEC fate far more efficiently than cells from alternative mesodermal protocols. 37 42 Following intrahepatic transplantation into neonatal NSG mice, these venous angioblasts generated mature, fenestrated LSECs with sinusoidal morphology, co-expressing CD31, CD32, and LYVE1, and exhibiting canonical scavenger functions such as *E. coli* uptake and acetylated-LDL binding. 37 In hemophilia A mice, transplanted iPSC-derived VECs matured into LSECs that secreted human F8 at levels sufficient to correct the severe bleeding phenotype without requiring additional F8 gene modification. 42 Longitudinal studies in FRG mice undergoing liver regeneration further showed that transplanted iPSC-derived ECs progressively repopulate the liver vasculature, contributing substantially to the sinusoidal network. 57 Single-cell RNA sequencing identified multiple endothelial clusters expressing LSEC markers including PECAM1, LYVE1, FCGR2B, CD36, and F8, with distinct subpopulations exhibiting zone 1, 2, and 3 LSEC-like signatures closely correlated with primary human liver endothelium. 57 Ultrastructural analysis confirmed the presence of fenestrations arranged in sieve plates, and functional assays demonstrated broad scavenger receptor activity against formaldehyde-treated albumin, acetylated-LDL, hyaluronic acid, and mannose-6-phosphate. 37 42 57

Most recently, entirely iPSC-derived sinusoidal vessels were generated by first specifying putative liver sinusoidal endothelial progenitors (iLSEPs) from CD32b ^+^ VECs exposed to BMP4, VEGF-A, and GSK3 inhibition, followed by maturation under high VEGF-A and TGF-β blockade. 39 In an inverted multilayered air–liquid interface (IMALI) three-dimensional culture, iLSEPs co-cultured with iPSC-derived hepatic endoderm (iHE) and septum transversum mesenchyme (iSTM) self-organized into human liver bud-like organoids (HLBOs). 39 These organoids developed CD32b ^+^ F8 ^+^ LYVE1 ^+^ sinusoidal-like networks that sprouted toward the oxygenated interface. 39 Transcriptomic analyses revealed progressive convergence of HLBO endothelial populations toward primary LSEC profiles, particularly zone ⅔ signatures. 39 HLBOs also generated peri-central GLS2 ^+^ GS ^+^ hepatocyte subsets and secreted multiple coagulation factors, including F8, over several weeks in culture. 39 WNT2 signaling from LSEC-like cells was critical for both hepatocyte maturation and endothelial branching; WNT2 knockdown diminished hepatocyte metabolic enzyme expression and impaired vascular network formation. 39 Upon transplantation into neonatal NSG mice, iLSEP-derived endothelium formed fully perfused, sinusoid-like vessels with functional connections to host circulation. 39 58 Collectively, these studies delineate a developmental trajectory from hybrid organoids containing primary vasculature to fully iPSC-derived, vascularized liver tissue capable of LSEC-specific functions, including fenestration, scavenging, coagulation factor production, and therapeutic correction of hemophilia A.

### Direct Reprogramming and Transdifferentiation

Direct reprogramming strategies bypass full pluripotency to generate ECs with enhanced expansion capacity and phenotypic stability compared with conventional iPSC differentiation ( Table 1 ). In one approach, brief transduction of human fibroblasts with OCT4, SOX2, KLF4, and c-MYC yields partial-iPS (PiPS) cells that efficiently adopt an endothelial fate through SETSIP-mediated activation of VE-cadherin, producing highly pure VE-cadherin ^+^ /VEGFR2 ^+^ populations without tumorigenic potential. 59 In another approach, lineage-committed c-Kit ^+^ amniotic cells are converted into mature rAC-VECs via transient ETV2 expression combined with sustained FLI1/ERG1 and TGF-β inhibition, rapidly generating nearly pure VE-cadherin ^+^ cells that expand to billions over weeks, exceeding yields achievable with standard iPSC-derived EC protocols. 60 In both systems, reprogrammed cells display genome-wide transcriptional profiles similar to adult ECs, including HUVECs and LSECs, form patent vascular networks in Matrigel and decellularized scaffolds, and integrate into regenerating liver sinusoids in vivo. 59 60

## Endothelial Cell Therapy Challenges

EC therapy faces substantial technical, biological, and translational challenges. Primary adult ECs, particularly LSECs, are difficult to obtain in sufficient numbers due to limited donor tissue and complex isolation procedures, while iPSC-derived ECs often exhibit restricted proliferation, lineage instability, and partial immaturity despite advances in differentiation protocols. 57 60 61 62 Experience from hepatocyte transplantation suggests that 5 to 10% liver repopulation may be required for therapeutic benefit; yet sustained engraftment at this level is rarely achieved, and clinical efficacy across metabolic indications remains inconsistent. 62 Cell engraftment is further constrained by cryopreservation challenges, as post-thaw viability and functional retention are variable, underscoring the need for robust, GMP-compliant preservation methods. 62 Current transplantation strategies often rely on harsh conditioning, such as monocrotaline, irradiation, or cyclophosphamide, to disrupt the sinusoidal barrier and create space for donor cells. Monocrotaline, in addition, carries oncogenic potential, and these regimens are not clinically acceptable. 37 38 Contaminating mesenchymal or fibroblast-like cells arising during differentiation must be rigorously removed to prevent ectopic matrix deposition or tumor-promoting effects, necessitating improved enrichment strategies and markers beyond pan-endothelial antigens like CD31, which cannot distinguish LSECs from other vascular subsets. 18 Safety concerns also encompass the tumorigenic potential of progenitor populations, immune rejection that may require prolonged immunosuppression even for ostensibly autologous products, and the logistical demands of patient-specific GMP manufacturing. 37 42 62 A key safety challenge is ensuring that transplanted healthy, angiogenic endothelial cells do not create a pro-tumor microenvironment that could promote hepatocarcinogenesis. 20 21 63 64 65 The molecular identity and optimal phenotype of therapeutic endothelial progenitors remain debated, including whether bone marrow–derived cells truly transdifferentiate into long-lived ECs or function primarily through paracrine trophic effect. 60 66 Clinical trials using heterogeneous endothelial or progenitor products have produced variable results, often without rigorous controls or long-term follow-up, and the overall therapeutic impact has been modest relative to investment. 51 67 Finally, most preclinical studies rely on immunocompromised mouse models (NSG, NSG-HA, FRG), which may not predict outcomes in immunocompetent hosts, and incomplete understanding of LSEC differentiation and the liver microenvironment complicates in vitro modeling and therapeutic optimization. 42 57 68

## Future Directions

Future LSEC-targeted therapies must overcome the lack of perfectly specific LSEC markers and Cre lines, which has restricted precise genetic and pharmacologic manipulation, 21 69 although the newly identified LSEC marker Oit3 may help address this challenge. 36 Promising strategies include maintaining or restoring LSEC differentiation via BMP9 and GATA4 signaling, re-establishing the VEGF/NO/sGC pathway using phosphodiesterase inhibitors or statins, and employing hedgehog pathway inhibitors such as tetramethylpyrazine to prevent or reverse capillarization. 21 52 Anti-angiogenic approaches that modulate the Ang2/Tie2 axis, angiotensin II signaling, or multi-kinase targets (e.g., sorafenib, lenvatinib) can exert both anti-inflammatory and anti-fibrotic effects. 21 Novel delivery systems are critical: nanoparticles that exploit LSEC mannose receptors have been used to deliver α-melittin, enhancing immune cell infiltration and NK cell maturation in liver metastasis models, while LSEC-targeted microRNA replacement (e.g., MiR-20a) has markedly reduced metastatic burden. 21 Precise tuning of Notch signaling also offers context-dependent opportunities, as Notch inhibition protects against capillarization and fibrosis but may favor hepatic metastasis, whereas Notch activation can restrict metastatic seeding of melanoma and colorectal cancer. 21

Human iPSC-derived LSECs represent a novel and promising avenue for cell-based liver therapies, with multiple differentiation protocols successfully generating functional LSECs capable of F8 production and therapeutic efficacy in preclinical models. 37 38 42 49 57 Critical optimization parameters include hypoxic culture, cyclic AMP agonism, and TGF-β inhibition, with venous rather than arterial pre-specification favoring LSEC marker expression, fenestration formation, and scavenger activity. 17 37 42 Outstanding translational challenges include developing clinically acceptable conditioning regimens that avoid MCT or irradiation, eliminating residual mesenchymal cells that could form fibrotic nodules, overcoming immune rejection via the use of hypoimmunogenic “universal donor” hPSC lines, and establishing GMP-compliant processes for large-scale manufacturing. 37 38 62 Early clinical experience with endothelial progenitor cell therapies in non-hepatic indications, such as pulmonary arterial hypertension, refractory angina, and critical limb ischemia, has shown limited but encouraging efficacy. 67 However, robust evidence for cardiovascular tissue repair remains scarce, suggesting that combination strategies with adjunct pharmacotherapies may be needed to unlock the full potential of EC-based interventions.

## Conclusion

LSECs constitute the liver's specialized fenestrated endothelium, orchestrating filtration, scavenging, and angiocrine signaling essential for liver homeostasis and regeneration. Acute injury rapidly disrupts LSEC fenestration and NO production, 20 25 27 while chronic injury drives capillarization, NO loss, and a shift toward profibrotic cues that activate HSCs and promote portal hypertension. 20 21 31 Preclinical studies indicate that transplantation of primary LSECs, EPCs, or iPSC-derived LSECs can restore sinusoidal architecture, improve hemodynamics, and correct endothelial-dependent metabolic deficits such as F8 deficiency. 12 19 To enable clinical translation, several critical challenges must be addressed: achieving truly LSEC-specific targeting, 36 54 developing safe conditioning regimens that avoid toxic pre-treatments, 21 66 establishing scalable and GMP-compliant production of venous-pre-specified iPSC-LSECs, 66 and designing rational combination strategies with antifibrotic or immunomodulatory therapies. 21 Collectively, these advances position EC therapy as a potentially transformative modality for patients with liver disease.

## Notes

Lay Summary Liver disease often begins when the tiny blood-filtering cells that line the liver's capillaries, liver sinusoidal endothelial cells (LSECs), lose their characteristic “window-like” openings. This endothelial cell (EC) dysfunction triggers scar formation, increases pressure in the portal vein, and impairs the liver's ability to heal and regenerate. Restoring a healthy endothelial layer may therefore interrupt these harmful cascades and help recover normal liver function. Researchers are now exploring cell-based therapies designed to replace or repair damaged LSECs. Potential approaches include transplantation of primary LSECs, stem cell–derived LSEC-like cells that can be generated at scale, and engineered progenitor cells delivered using biomaterial scaffolds. Early animal studies suggest that introducing functional ECs into the liver can reduce fibrosis, lower portal pressure, and improve overall liver function. By shifting the focus from managing symptoms to rebuilding the liver's vascular architecture, this emerging strategy offers a promising new therapeutic avenue for patients with liver disease.
